# Non-invasive prenatal testing (NIPT): a better option for patients

**DOI:** 10.1186/1755-8166-7-S1-I17

**Published:** 2014-01-21

**Authors:** Ashish Fauzdar

**Affiliations:** 1 Quest Diagnostics India Private Limited, A-17 Info City, Sector 34, Gurgaon, Haryana, India

## 

At present there are two methods to determine the chromosome health of unborn fetus in pregnant women. One is through maternal serum screening that is considered as simple but less sensitive method and the second options involves invasive methods like amniocentesis or chorionic villus sampling (CVS) for women who are found to be positive in maternal serum screening. Recent studies have demonstrated that non-invasive prenatal testing (NIPT) using cell-free fetal DNA (cfDNA) present in circulating maternal blood is considered as one the most effective method of screening for trisomy 21.

The single nucleotide polymorphism (SNP) based Non-invasive Pre-natal test, being offered by Quest Diagnostics in technical collaboration with Natera Inc to determine chromosomal copy number by looking for specific patterns of (SNPs). This second generation technology analyzes cfDNA in a single reaction targeting 19,500 single nucleotide polymorphisms which are the most informative portions of an individual’s DNA selected across all the analyzed chromosomes.

Two published study demonstrated in the women undergone both invasive chorionic villus samplings followed by karyotyping in conjunction with non-invasive prenatal testing the efficacy of test. Data from Nicolaides *et al*., 2013 paper on total of 242 cases that include thirty two cases aneuploidy included trisomy 21 (n=25) which were correctly identified with 100% sensitivity and 100% specificity with no false positive and false negative. Another study by Zimmerman *et al*., 2012 study on 166 samples from pregnant women, including 11 trisomy 21, three trisomy 18, two trisomy 13, two 45, X, and two 47,XXY samples including 146 low risk pregnancies. The study also correctly reported the chromosome copy number of all five chromosomes in 145 samples that passed a DNA quality test.

The studies had demonstrated that SNP based NIPT is considered as an effective method of screening for common chromosomal abnormalities with detection rate of more than 99% and false positive rate of less than 0.1%. The initial finding shows NIPT approach has potential to avoid invasive procedure and can be provided an option to high risk pregnancies i.e. those with advanced maternal age, screens positive for biochemical screening and history of pregnancies with previous aneuploidies.

